# Transcriptional Activation of Pyoluteorin Operon Mediated by the LysR-Type Regulator PltR Bound at a 22 bp *lys* Box in *Pseudomonas aeruginosa* M18

**DOI:** 10.1371/journal.pone.0039538

**Published:** 2012-06-25

**Authors:** Sainan Li, Xianqing Huang, Guohao Wang, Yuquan Xu

**Affiliations:** State Key Laboratory of Microbial Metabolism and School of Life Sciences & Biotechnology, Shanghai Jiao Tong University, Shanghai, People's Republic of China; Belgian Nuclear Research Centre SCK/CEN, Belgium

## Abstract

*Pseudomonas aeruginosa* M18, a rhizosphere-isolated bacterial strain showing strong antifungal activity, can produce secondary metabolites such as phenazine-1-carboxylic acid and pyoluteorin (Plt). The LysR-type transcriptional regulator PltR activates the Plt biosynthesis operon *pltLABCDEFG*, the expression of which is induced by Plt. Here, we identified and characterized the non-conserved *pltL* promoter (*pltLp*) specifically activated by PltR and its upstream neighboring *lys* box from the complicated *pltR–pltL* intergenic sequence. The 22 bp palindromic *lys* box, which consists of two 9 bp complementary inverted repeats interrupted by 4 bp, was found to contain the conserved, GC-rich LysR-binding motif (T-N_11_-A). Evidence obtained *in vivo* from mutational and *lacZ* report analyses and *in vitro* from electrophoretic mobility shift assays reveals that the PltR protein directly bound to the *pltLp* region as the indispensable binding motif “*lys* box”, thereby transcriptionally activating the *pltLp*-driven *plt* operon expression. Plt, as a potential non-essential coinducer of PltR, specifically induced the *pltLp* expression and thus strengthened its biosynthetic *plt* operon expression.

## Introduction

Pyoluteorin (Plt) is a *Pseudomonas*-produced antifungal compound with a resorcinol ring linked to a bichlorinated pyrrole moiety produced by a polyketide synthase–non-ribosomal peptide synthetase (PKS/NRPS) hybrid biosynthetic pathway [1]. The Plt biosynthetic gene cluster, which is approximately 32 kb long, has been cloned and characterized in *Pseudomonas fluorescens* Pf-5 [2], [3] and *P. aeruginosa* M18 [4], [5], and identified through complete genome sequencing in *P. aeruginosa* LESB58 [6]. The *plt* locus shows a high level of conservation in gene organization and size among these three *Pseudomonas* strains. *P. aeruginosa* M18 shares 99% nucleotide sequence identity in the *plt* gene locus with LESB58 [6], [7], but shows a certain degree of difference in nucleotide sequence, especially for the non-coding sequence from Pf-5 [8]. Intriguingly, strain LESB58 cannot produce Plt [9] because of a frameshift mutation in the *pltB* gene [6]. The *plt* gene cluster is composed of two pairs of divergently transcribed operons: one pair is the *pltLABCDEFG* and *pltRM* operons responsible for Plt biosynthesis and its activation, the other pair is the *pltHIJKNO* and *pltZ* operons involved in Plt export and its repression. However, the *plt* gene cluster has not yet been experimentally dissected for the *pltR* and *pltL* intergenic non-coding region (485 bp for Pf-5 and 652 bp for M18) containing a number of promoters and *cis*-acting elements in two opposite directions [2]–[5].

Plt biosynthesis is regulated by the Gac/Rsm regulatory cascade, a ubiquitous global regulatory pathway that consists of the two-component signal transduction system GacS/GacA, the sRNAs RsmY and RsmZ, and the RNA-binding repressor protein RsmA repressing access to the ribosome binding site (RBS) [10]–[13]. Our previous studies have also shown that Plt biosynthesis is strongly repressed by three quorum sensing (QS) systems, namely, LasI/LasR, RhlI/RhlR, and PQS/PqsR, in *P. aeruginosa* M18 [14]–[16]. Aside from these global regulatory systems, the pathway-specific regulator PltR, a typical LysR-type transcriptional regulator (LTTR), is responsible for the transcriptional activation of the *pltLABCDEFG* biosynthetic operon [3], [17]. Expression of the *plt* operon is induced by Plt [4], [18]. In addition, the TetR-type regulator PltZ negatively regulates the divergently transcribed *pltHIJKNO* ABC (ATP-binding cassette) export operon and, thus, indirectly downregulates Plt production [4], [5]. However, the molecular mechanism underlying the transcriptional activation and autoinduction of the *plt* biosynthetic operon, for example, PltR's target DNA motif, Plt's receptor protein, and the effect of Plt on PltR–DNA interaction, remains unknown.

The LTTR family represents one of the largest classes of bacterial transcriptional regulatory proteins [19], [20]. Typically, the conservative LTTR protein comprises a helix-turn-helix (HTH) DNA-binding motif at the N-terminus and a cofactor-binding motif at the C-terminus. LTTRs are mostly characterized as transcriptional activators of a single divergently transcribed gene or operon and negative autoregulators [19]. With the continuous identification of large numbers of LTTRs, some of them have been extended to function as global transcriptional activators or repressors of unlinked genes or operons involved in metabolism, QS, virulence, and so on. These LTTRs include PqsR, a response regulator in the PQS (*Pseudomonas* quinoline signal)-mediated QS system [14], [21]. Aside from repressing Plt biosynthesis, PqsR can also activate the phenazine-1-carboxylic acid (PCA) biosynthesis in *P. aeruginosa* M18 [14]. Two LTTRs with opposite effects on the *plt* operon, PltR and PqsR, likely differ in molecular regulatory mechanisms. The mechanisms of both remain uncharacterized.

Autoinduction of microbiological metabolism, including the QS signal and antibiotic biosynthesis, is widespread in the microbial community. The *N*-acyl homoserine lactones (AHLs) in Gram-negative bacteria typically induce the expression of their own LuxI-type biosynthetic genes in collaboration with the LuxR-type response regulators [22]. Aside from the autoinduction of Plt biosynthesis involved in the present study, an increasing number of secondary metabolites produced by *Pseudomonas* spp., including 2,4-diacetylphloroglucinol [23], PCA [24], and pyoverdine [25], have been shown to induce their own biosynthesis in a concentration-dependent manner.

The current study identifies three promoters in the *pltL* direction, including the non-conserved *pltL* promoter (*pltLp*) specifically activated by PltR, and one *pltR* promoter in the *pltR* direction within the intergenic region between the divergently transcribed *pltL* and *pltR* genes. The LTTR PltR directly binds to the *pltLp* region at the indispensable and intact 22 bp palindromic *lys* box and, thus, specifically activating *plt* operon expression. Plt, as a potential and non-essential cofactor of PltR, specifically induces the *pltLp* and thus strengthens the *plt* operon expression.

## Results

### Identification of the promoter in the intergenic region between divergently transcribed *pltL* and *pltR* genes

**Figure 1:**
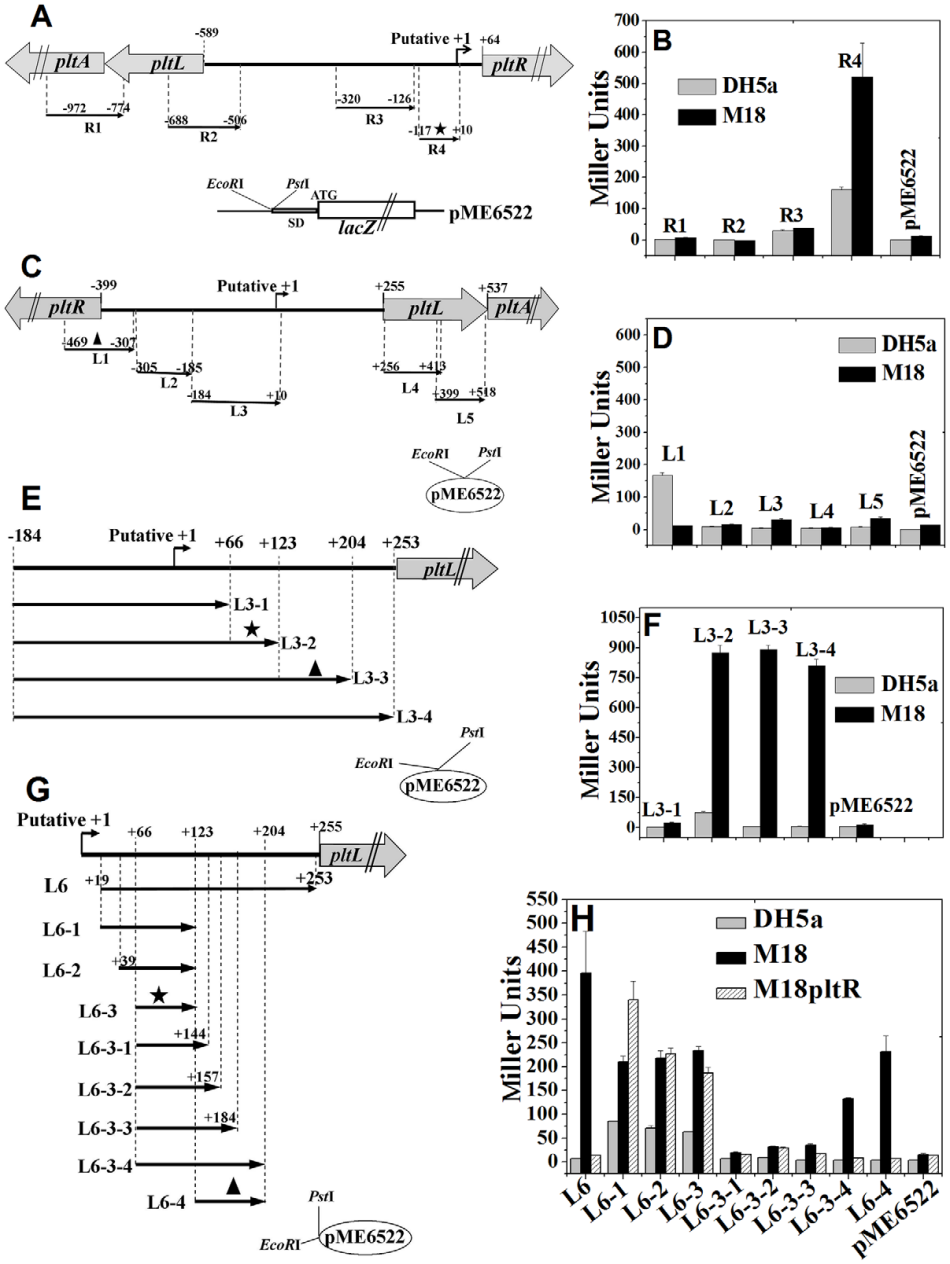
Detection of promoter activity in the intergenic region between divergently transcribed *pltR* and *pltLABCDEFG* genes using the *lacZ* reporter gene. (**A** and **B**) Promoter detection in the *pltR* direction. (A) Four fragments, R1 to R4, containing five putative promoters in the *pltR* direction (predicted by the NNPP promoter online prediction with a score cutoff 0.80) were fused with the promoterless *lacZ* gene on the plasmid pME6522. The R3 fragment includes two putative promoters. The putative +1 site indicates the putative TSS of the fifth predicted promoter. (B) β-Galactosidase expression (Miller Units) from above these *lacZ* fusion plasmids was assayed in both *E. coli* DH5α and *P. aeruginosa* M18 after 15 h of growth in KMB. The R4 fragment was detected to include a true promoter (shown by an asterisk). (**C** and **D**) Promoter detection in the *pltLABCDEFG* operon direction. (C) Five fragments, L1 to L5, which contain five putative promoters in the *pltL* direction, were respectively fused into the upstream of the promoterless *lacZ* gene on pME6522. Putative +1 indicates the putative TSS of the third predicted promoter. (D) β-Galactosidase expression from the above *lacZ* fusion recombined plasmids was measured in both DH5α and M18. The L1 fragment (marked with a triangle) exhibited a certain degree of promoter activity in DH5α, but not in M18. The other four fragments, L2 to L5, did not show any evident promoter activity. (**E** and **F**) Detection of promoter or its elements by prolongation analysis within the DNA fragment spanning from +10 to the *pltL* ATG, which was not predicted to carry promoters. (E) Four prolonging fragments based on L3, namely, L3–1 to L3–4, were respectively fused with the *lacZ* gene in pME6522. (F) The resulting *lacZ* fusion plasmids were assayed for β-galactosidase expressions in both DH5α and M18. The region covering +66 to +124 bp (relative to the putative +1 site) contained either a stronger promoter or its elements (marked with an asterisk). The *L3–3–lacZ* fusion expression significantly decreased, compared with that in the *L3–2–lacZ* fusion expression in *E. coli* DH5α, which implies that the promoter located at +66 to +124bp is not likely *pltLp* activated by PltR. Moreover, the region from +124 to +205 bp (marked with a triangle) possibly contains another promoter under the transcriptional activation of PltR. (**G** and **H**) Distinguishing *pltLp* activated by PltR from other promoters by deletion analysis. (G) Four fragments (L6 and its shortened derivative fragments, namely, L6–1 to L6–3), four continuously prolonged fragments based on L6–3 (L6-3-1 to L6-3-4), and the L6–4 fragment, were respectively cloned into pME6522. The L6 fragment was not predicted (score cutoff 0.60) to contain putative promoter regions. (H) The β-galactosidase expression from these recombinant *lacZ* reporter plasmids was analyzed in *E. coli* DH5α, *P. aeruginosa* M18, and the *pltR* mutant M18pltR. An intact promoter contained in the region from +66 to +124 (marked with an asterisk) was not controlled by PltR. The *pltLp* activated by PltR was located from +124 to +205 bp (marked with a triangle), relative to the putative +1 site.

In the Plt biosynthetic structural, regulatory, and transport gene cluster of *P. aeruginosa* M18 (Genbank accession number AY394844), the divergently transcribed *pltL* and *pltR* genes were separated by a large and complex intergenic region (652 bp) that likely contains a number of promoters and regulatory elements. Generally, putative promoters predicted by the NNPP software (*P*>0.8) need further experimental confirmation through *lacZ* reporter fusion analysis. The NNPP analysis showed five putative promoters in both the *pltR* and *pltL* directions. These predicted promoter fragments were respectively amplified and fused with the promoterless *lacZ* gene in the plasmid pME6522 (Fig. 1A and 1C). β-Galactosidase activity expressed from these putative promoter–*lacZ* fusion plasmids were assayed in *E. coli* DH5α and *P. aeruginosa* M18. As shown in Fig. 1B, among the *R1* to *R4–lacZ* fusion plasmids that carried five putative promoters in the *pltR* direction (the R3 fragment contains two putative promoters), only the *R4*–*lacZ* fusion expressed a significantly higher β-galactosidase activity in both DH5α and M18. The *R1* to *R3*–*lacZ* fusions did not display noticeable differences in β-galactosidase expression from the empty plasmid pME6522 as the control. The result indicates that the R4 fragment, spanning from−116 bp to +10 bp relative to the putative TSS (transcriptional start site, +1) of *pltR*, harbors an actual *pltR* promoter that also shows a certain level of basal expression activity in *E. coli* DH5α.

Similarly, five predicted promoter fragments (L1 to L5) in the *pltL* direction were respectively fused with the *lacZ* gene of pME6522 (Fig. 1C). Two of the predicted promoters extended into the *pltL* ORF. In the M18 strain, all five putative promoters did not show any significant expression compared with the pME6522 control (Fig. 1D). However, the *L1–lacZ* fusion showed a relatively higher expression (approximately 160 Miller units) in *E. coli* DH5α, but was almost entirely inhibited in *P. aeruginosa* M18. This occurrence is probably due to the presence of negative specific repressors in M18. The 164 bp L1 fragment was partially overlapped with the *pltR* promoter (R4) and showed similar levels of promoter activity, implying that this region likely contains a bidirectional promoter.

With the highest score of 0.94 in the NNPP promoter prediction, the putative promoter contained in the L3 fragment was supposed to be the most probable *pltL* promoter. However, no promoter activity was detected in the L3 fragment by the above *lacZ* fusion analysis (Fig. 1D). For this, four fragments that continuously extended from the right side of L3, namely, L3–1 to L3–4, were respectively cloned into the plasmid pME6522 to construct the corresponding *lacZ* fusion reporter plasmids (Fig. 1E). The results of the β-galactosidase assay in the M18 strain showed that the *L3–2–lacZ* fusion displayed stronger expression (approximately 900 Miller units) in the presence of the fragment ranging from +66 bp to +124 bp relative to the putative *+*1 site (Fig. 1F), implying that this 59 bp fragment likely contains strong promoters or additional activating *cis*-regulatory motifs. In addition, the *L3–2–lacZ* fusion also expressed approximately 70 Miller units of β-galactosidase activity in *E. coli* DH5α. The *L3–3–lacZ* fusion carrying an additional extended sequence showed similar expression levels of β-galactosidase as the *L3–2–lacZ* fusion in the M18 strain. However, *L3–3–lacZ* fusion expression was inhibited to the level of the control plasmid in DH5α (Fig. 1E). These results suggest that the promoter activity expressed by the fragment ranging from +66 bp to +124 bp is probably independent of the transcriptional activator PltR and inhibited by the presence of one inhibitory motif within the region from +124 to +205 bp (relative to the putative +1). Therefore, the search for the PltR-activated *pltLp* in the +124 to +205 bp region was continued.

Based on the *L3–1* to *L3–4–lacZ* fusion analysis results (Fig. 1E), the 235 bp L6 fragment (+19 bp to +253 bp) (Fig. 1G), which was not predicted by NNPP (even above a minimum promoter score of 0.6) to carry any promoters, could be speculated to actually harbor strong promoters. As expected, the *L6–lacZ* fusion showed stronger expression activity in the M18 strain (Fig. 1H). In addition, the expression of *L6–lacZ* fusion was almost entirely inhibited in the *pltR* mutant M18pltR (Fig. 1H), suggesting that one promoter contained in the L6 fragment is dependent on PltR. To narrow down the key promoter region, three *lacZ* fusion plasmids, which respectively contains three truncated fragments L6–1, L6–2 and L6–3, were constructed (Fig. 1G). These three *lacZ* fusions all expressed relatively higher levels of β-galactosidase activity in *E. coli* DH5α, *P. aeruginosa* M18, and M18pltR (Fig. 1H). Therefore, the L6–3 fragment ranging from +66 to +124 bp contains an intact PltR-independent promoter that may be a non-*pltLp* promoter.

Based on the differential expression between the *L6–lacZ* and *L6–1*–*lacZ* fusions in both *E. coli* DH5α and *P. aeruginosa* M18pltR (Fig. 1H), as well as the differential expression between the *L3–2–lacZ* and the *L3–3–lacZ* fusions in DH5α (Fig. 1F), the fragment from +124 bp to +205 bp contains a potential promoter activated by PltR. Another set of *lacZ* fusion plasmids, which contains four continually prolonged fragments L6-3-1 to L6-3-4 based on L6–3, were constructed and assayed for β-galactosidase expression in strains DH5α, M18, and M18pltR (Figs. 1F and 1H). Interestingly, the relatively higher level of *L6–3–lacZ* fusion expression was almost entirely inhibited by a short sequence prolongation (+124 bp to +143 bp) in all three strains. This inhibited expression in the *L6-3-1* to *L6-3-3–lacZ* fusion was reversed to the greatest extent only in M18 when the L6-3-4 fragment extended to +205 (i.e., L6-3-4) (Fig. 1H). In addition, the *L6–4–lacZ* fusion expression was almost entirely abolished in both DH5α and M18pltR (Fig. 1H). These results strongly suggest that the *pltLp* activated by PltR is located from +124 to +205 bp. Moreover, this region was also shown to carry an inhibitory *cis*-element (+124 bp to +143 bp) for the upstream promoter contained in the L6–3 fragment covering from +66 bp to +124 bp relative to the putative *pltL* +1 site.

### Mapping the TSSs of *pltL* and *pltR*

**Figure 2:**
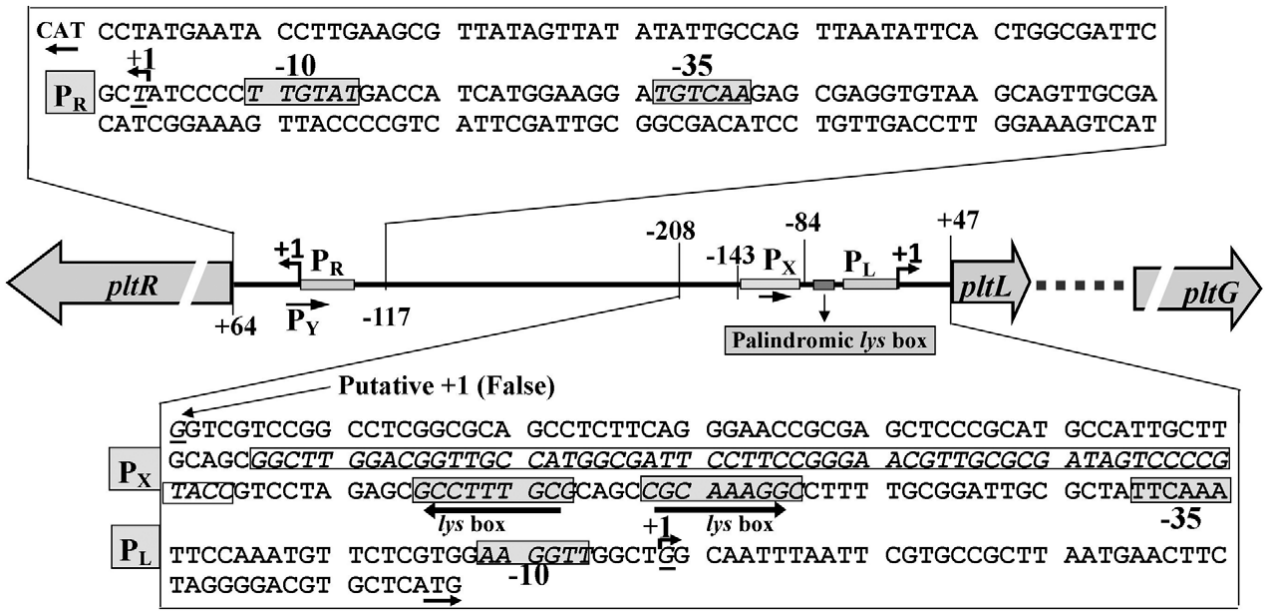
Mapping the TSSs and promoters of *pltR* and *pltL* genes through 5′RACE and the above *lacZ* reporter analysis (Fig. 1). P_R_, the *pltR* promoter; P_L_, the *pltLp* which needs to be specifically activated by PltR; P_X_, a non-PltR controlled promoter closely neighboring the upstream of P_L_; P_Y_, another potential promoter silent in *P. aeruginosa* M18, but active in *E. coli* DH5α. The TSS (+1) of the *pltR* transcript was located at 63 bp upstream of the *pltR* translational start codon ATG, which is identical to the predicted +1 site. The TSS of the *plt* operon was located at 46 bp upstream of the *pltL* ATG. Closely linked to the *pltLp* with the P_x_ promoter, a palindromic *lys* box, including two 9 bp inverted complementary sequences separated by 4 bases, is boxed and highlighted by two head-to-head arrows.

To better define the promoter region and *cis*-elements responsible for the regulation of *plt* operon expression, the TSS (+1) of the *pltL* and *pltR* genes were mapped using 5′RACE (5′Rapid amplification of cDNA end). The results are shown in Fig. 2. The actual +1 site of *pltR*, which is identical to the putative +1 site, was located at 63 bp upstream from the *pltR* translational start codon ATG (Fig. 2). The actual *pltR* promoter identified by 5′RACE was basically consistent with the putative *pltR* promoter (predicted by NNPP) contained in the R4 fragment (Fig. 1A).

Relative to *pltR*, the composition of the promoters and regulatory elements in the *pltL* direction were more complex and diverse. The 5′RACE result shows that the *pltL* TSS is located 46 bp upstream from the *pltL* ATG site (Fig. 2). The L6 fragment (Fig. 1G), which was not predicted by NNPP to carry any promoter, actually contains two promoters, namely, the *pltLp* activated by PltR and its neighboring upstream promoter (P_x_). The putative −10 and −35 regions in these promoters differ substantially from each other and are also markedly different from the canonical −10 (TTGACA) and −35 (TATAAT) sequences. As positively activated genes or operons generally have poor −35 and/or −10 regions their transcription mostly relies on the assistance of an activator protein to stabilize promoter-RNAP interaction. The fact that PltR regulates the *pltL* promoter but not the P_x_ promoter is consistent with the low level of conservation between the −10 and −35 regions of the two promoters. The P_x_ promoter was located at the −143 bp to −86 bp relative to the *pltL* TSS (Fig. 2), which corresponds to the L6–3 fragment (Fig. 1G). However, several attempts to experimentally identify the TSS of the P_x_ promoter failed. Remarkably, a perfect palindromic motif composed of two 9 bp reverse complementary sequences separated by 4 bp was sandwiched exactly between the pltL and P_x_ promoters (Fig. 2). The preceding results show that the presence of half of this palindromic motif could abolish the expression of the P_x_ promoter (Figs. 1G and 1H, L6-3-1). Furthermore, the 22-bp interrupted palindromic motif perfectly matched the conservative LTTR binding site (T-N_11_-A) [19]. Therefore, this motif, designated as the *lys* box hereafter, was supposed to function as the target site of the LTTR protein PltR, which was verified by the following experiments.

### The *pltLp* promoter region (−84 bp to +1) was directly bound and activated by PltR

**Figure 3:**
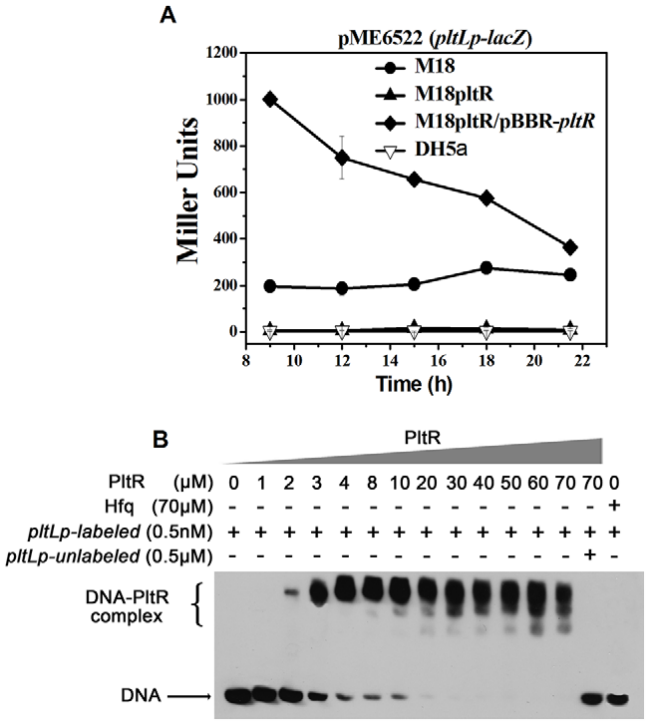
PltR transcriptionally activated *pltLABCDEFG* operon expression *via* direct binding to the *pltLp* region from −84 bp to +1. (**A**) Transcriptional activation of *pltLp* by PltR. The β-galactosidase expression (Miller Units) from *pltLp–lacZ* transcriptional fusion (p6522–pltLp) was assayed in M18, M18pltR, M18pltR/pBBR–pltR, and DH5α. The *pltLp*–*lacZ* expression was entirely inhibited in the *pltR* mutant M18pltR, which, in turn, was reversed and even enormously enhanced by the exogenous *pltR* overexpression from the plasmid pBBR–pltR. (**B**) Direct binding of PltR to *pltLp*, assessed by EMSA. 0.5 nM biotin-labelled *pltLp* promoter fragment (−84 bp to +1) were respectively incubated with increasing amounts of His–PltR protein (0 to 70 μM, Lane 1 to 13). For competition reactions, 0.5 μM unlabeled *pltLp* fragment was included in the binding reaction in molar as shown in Lane 14. The Hfq protein was used as a negative control (Lane 15).

Among the two neighboring promoters (*pltLp* and P_x_) in the *pltL* direction, P_x_ was not controlled by PltR (Figs. 1G and 1F). The next focus was on characterizing the PltR-activated *pltLp*. To confirm the activation function of PltR on the experimentally positioned *pltLp*, the *pltLp–lacZ* transcriptional fusion plasmid (p6522–pltLp) containing the *pltLp* and its upstream palindromic *lys* box (−84 bp to +1), was constructed and transformed into *E. coli* DH5α, *P. aeruginosa* M18, the *pltR* mutant M18pltR and its complementary strain M18pltR/pBBR–pltR. The β-galactosidase activity was measured in KMB to compare *pltLp–lacZ* fusion expression between the presence and absence of *pltR*. As shown in Fig. 3A, the expression of the *pltLp–lacZ* fusion was completely inhibited in M18pltR when compared with that (approximately 200 Miller units) in the wild-type strain M18. With the introduction of the *pltR* overexpression plasmid pBBR–pltR, the *pltLp–lacZ* expression in the *pltR* mutant was restored and even significantly increased, compared with that in the M18 strain. The result clearly demonstrates that the 85 bp promoter region comprising its neighboring *lys* box needed to be activated by PltR (Fig. 3A). In addition, no evident expression in the *pltLp–lacZ* fusion was detected in *E. coli* DH5α (Fig. 3A). The *pltLp–lacZ* expression was not restored in the DH5α strain harboring the double plasmids, pBBR–pltR, and p6522–pltLp (data not shown), indicating that the *pltLp* expression requires the participation of other activation factors aside from the indispensable activator PltR.

EMSA was further designed to detect whether PltR directly binds to the *pltLp* region. The *pltLp* fragment (−84 bp to +1) was chosen to assess its binding affinity with PltR. As presented in Fig. 3B (Lanes 1 to 13), when 0.5 nM biotin-labeled *pltLp* fragments were incubated with increasing amounts of PltR protein (0 to 70 μM) in a 10 μl reaction system, the PltR protein showed specific binding activity with *pltLp*. Accompanied by the increasing concentration of PltR, the lagged bands representing the PltR-*pltLp* complex gradually strengthened while the biotin-labled free DNA probes significantly declined and was almost entirely bound by 20 μM PltR. The Hfq protein, as negative control, showed no binding activity with *pltLp*. A 1000-fold molar excess of unlabeled *pltLp* probes entirely displaced the biotin-labeled probes from PltR. Based on these data, it can be concluded that PltR directly binds the *pltLp*, thereby activating *plt* operon expression. This observation deserves further investigation to define the key sequence responsible for the binding of PltR to *pltLp*.

### The 22 bp palindromic *lys* box is indispensable to the binding and activation of the *pltLp* by PltR

**Figure 4:**
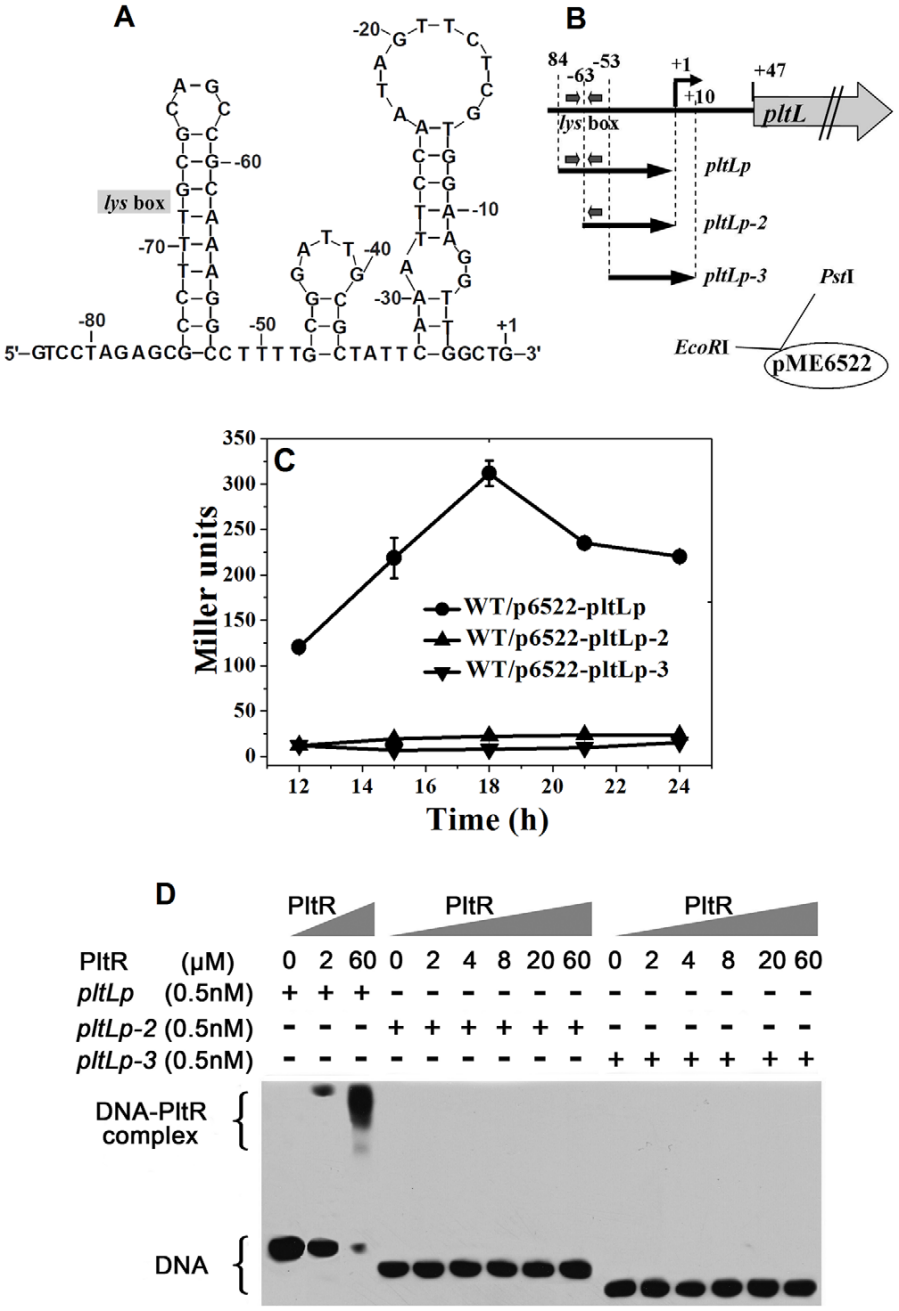
The 22 bp lys box (−74 to −53 bp) neighboring upstream of pltLp was indispensable to the transcriptional activation of PltR on the plt operon and the direct binding of PltR to pltLp. (**A**) The putative DNA secondary structure (by MEME) of the pltLp region (−84 bp to +1). (**B**) The *pltLp* fragment (*pltLp*, −84 bp to +1) and two truncated fragments with a half or entire deletion of the palindrome motif (*pltLp-2* and *pltLp-3*) were respectively fused with the *lacZ* reporter gene in pME6522. The palindrome *lys* box is shown with two thick arrows. (**C**) β-Galactosidase expression (Miller Units) from three *lacZ* fusion plasmids (p6522–pltLp, pltLp-2, pltLp-3) was assayed in the M18 strain. A half or entire deletion of the palindrome motif resulted in complete inhibition of the *pltLp*–*lacZ* expression in the M18 strain. (**D**) Direct binding of PltR to *pltLp* was eliminated by a half or entire deletion in the palindrome *lys* box. 0.5 nM *pltLp* and two truncated fragments (*pltLp-2* and *pltLp-3*) were respectively incubated with increasing amounts of His–PltR protein.

A putative secondary structure folded from *pltLp* and its upstream palindromic box (−84 bp to +1) by Mfold 3.2 is shown in Fig. 4A. The 22 bp *lys* box (−74 bp to −53 bp) constituting a typical T-N_11_-A LTTR binding site was supposed to form into a stem loop structure for binding and activation by PltR. Another two stem loops located from −47 bp to −4 bp were assumed as the *cis*-elements (−10 and −35 sequences) for RNA polymerase. Two *pltLp–lacZ* derivative plasmids, p6522–pltLp-2 and p6522–pltLp-3, which respectively carry a deletion of half or the entire *lys* box, were constructed to assess the effect of the *lys* box on *pltLp* activity (Fig. 4B). As shown in Fig. 4C, half and entire deletion of the *lys* box induced complete inhibition of the *pltLp–lacZ* fusion expression to approximately the expression level of the empty plasmid pME6522. Without the presence of the complete *lys* box, the *pltLp* could not be initiated. This confirms that the *lys* box is indispensable for the initiation and activation of the *pltLp*.

Correspondingly, the biotin-labeled *pltLp*, *pltLp-2*, and *pltLp-3* DNA probes (0.5 nM) were respectively utilized to carry out the EMSA analysis with increasing PltR concentrations (0 to 60 μM). The results are shown in Fig. 4D. The PltR protein displayed stronger binding affinity with the *pltLp* fragment with the intact *lys* box. As expected, half or whole deletion of the *lys* box completely abolished the binding of *pltLp* with PltR. No evident binding complexes were observed in the EMSA between the biotin-labeled *pltLp-2* or *pltLp-3* probe and the PltR protein (Fig. 4D, Lanes 4 to 9 and Lanes 10 to 15). The results suggest that PltR directly binds to the *pltLp* region at the indispensable and intact palindromic *lys* box and thus activate the *pltLp* expression.

### Effects of point mutations in the *lys* box on the expression and PltR binding of *pltLp*

**Figure 5:**
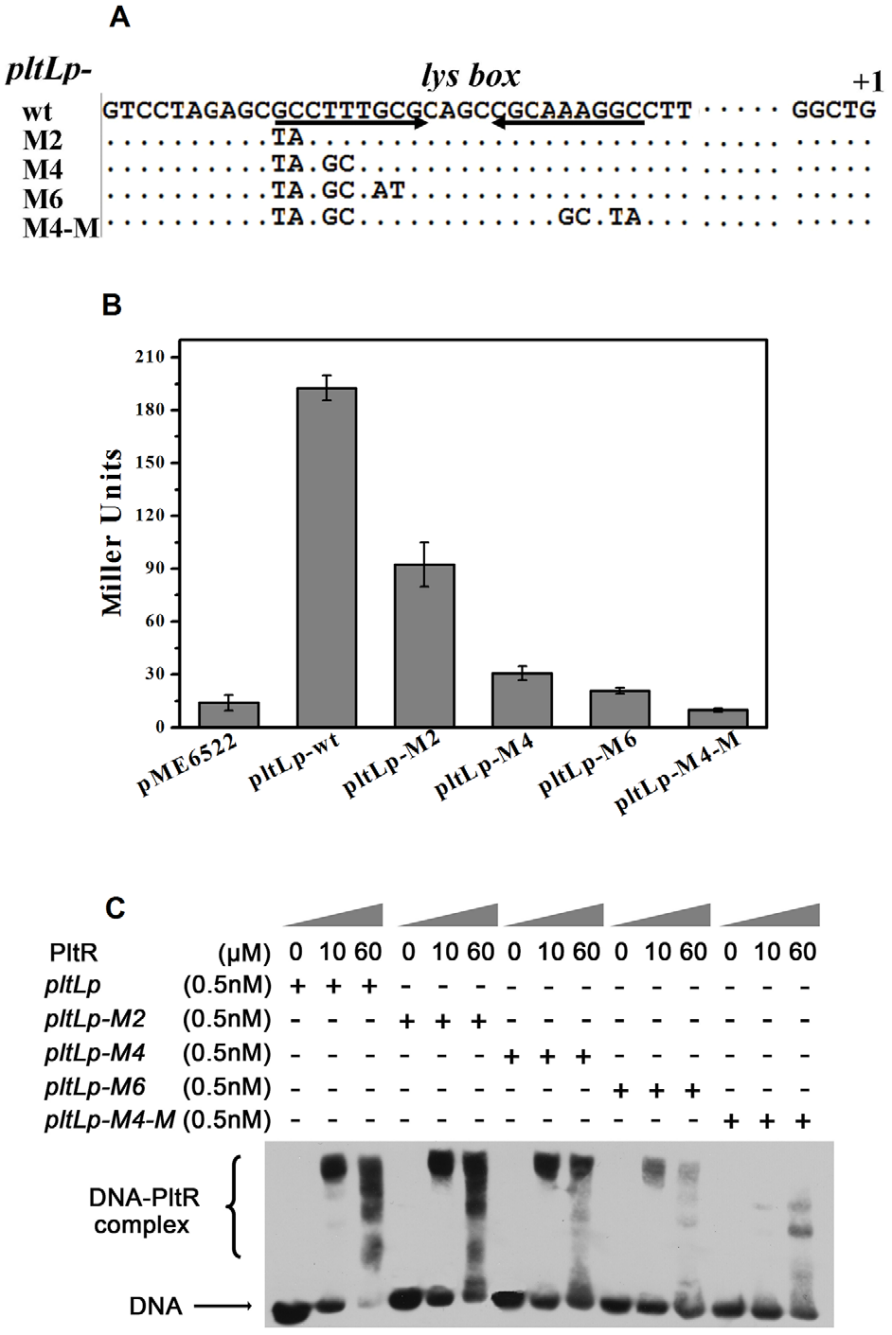
Effects of *lys* box mutations on *pltLp* expression and the interaction between PltR and *pltLp*. (**A**) Construction map of the mutagenized *pltLp* with *lys* box mutations. Three *pltLp* mutant fragments, *pltLp-M2*, *-M4*, and *-M6*, respectively carried 2 bp, 4 bp, and 6 bp replacements in the *lys* box. The *pltLp* mutant M4-M was constructed by introducing a 4 bp complementary replacement into the *pltLp-M4* mutant. The above four *pltLp* mutagenized fragments were respectively cloned into pME6522. (**B**) β-galactosidase expression (Miller Units) from the above *lacZ* fusion plasmids was measured in the M18 strain. The *pltLp* expression gradually and significantly decreased, even entirely inhibited, with the opening of the *lys* box formed stem loop (Fig. 4A) by base substitutions. (**C**) The binding of PltR to *pltLp* was significantly weakened and even eliminated by the *lys* box mutations. *pltLp* and its derivative mutated fragments (0.5 nM) were respectively incubated with increasing amounts of PltR protein. The concentration of PltR was 0, 10, and 60 μM, respectively.

To determine the effects of the *lys* box mutations on *plt* operon expression and PltR–*pltLp* binding, four *pltLp* derivative fragments carrying a serial of point mutations in the *lys* box were amplified and used to construct *lacZ* fusion plasmids and carry out an EMSA analysis. To open the putative stem-loop structure formed by the *lys* box (Fig. 4A), 2 bp (M2), 4 bp (M4), and 6 bp (M6) substitutions were designed in one of two 9 bp reversely complementary sequences in the *lys* box (Fig. 5A). The other 4 bp replacement was introduced to the M4 mutant to be complementary to the originally replaced 4 bp, producing the M4-M *pltLp* mutant, which could regenerate a 22 bp stem-loop structure with different base arrangements from the wild-type *pltLp*. The β-galactosidase activity expressed from the *pltLp–lacZ* fusion wild-type plasmid (p6522–pltLp) and its four derived mutant plasmids were assayed in *P. aeruginosa* M18. As depicted in Fig. 5B, the *pltLp–lacZ* fusion expression was significantly reduced, even entirely abolished, by the four *lys*-box mutants (Fig. 5B). The β-galactosidase expression of *pltLp–lacZ*, *pltLp*-*M2*–*lacZ*, *pltLp*-*M4*–*lacZ*, to *pltLp*-*M6*–*lacZ* fusion, showed a gradual and significant decreasing trend with the gradual opening of the stem-loop structure, which suggests that the secondary structure of the *lys* box plays a key role in the binding and activation of the *pltLp* by PltR. In addition, the inhibited β-galactosidase expression from the *pltLp-M4*–*lacZ* fusion was not restored; it continued to decrease to the expression level of the empty plasmid pME6522 by a complementary 4 bp substitution carried by the M4-M mutant. This finding implies that the sequence specificity of the *lys* box is also essential for the binding and activation of the *pltLp* by PltR.

Biotin-labeled DNA probes corresponding to the wild-type and mutagenized fragments of *pltLp* were subjected to an EMSA to assess the influence of point mutations in the *lys* box on the PltR–*pltLp* binding interaction. Subsequently, 0.5 nM biotin-labeled DNA probes were co-incubated with 0, 10, and 60 μM PltR. As shown in Fig. 5C, a 2 bp (or 4 bp, 6 bp) substitution and an 8 bp complementary substitution in the *lys* box resulted in significant decrease in the *pltLp* binding activity by PltR. The decrease was clearly reflected by an enormous reduction in the intensity of the retarded PltR–*pltLp*-M2 (or M4, M6, M4-M) complex bands, compared with those of the PltR–*pltLp*–wt complex bands. With the number of substituted bases increasing from 2 bp to 6 bp, the stem-loop structure formed by the *lys* box was opened step by step and thus the binding between the DNA probes and PltR gradually declined. Although the stem-loop structure was reshaped by a 4 bp complementary substitution in *pltLp–M4-M*, its binding with PltR was not restored to levels exceeding that of *pltLp-M4* with PltR. This experiment confirms that the secondary structure and the primary sequence of the *lys* box are crucial for the PltR-activated expression of the *plt* operon.

### Plt specifically induce the *pltL* promoter and thus enhance the *plt* operon expression

**Figure 6:**
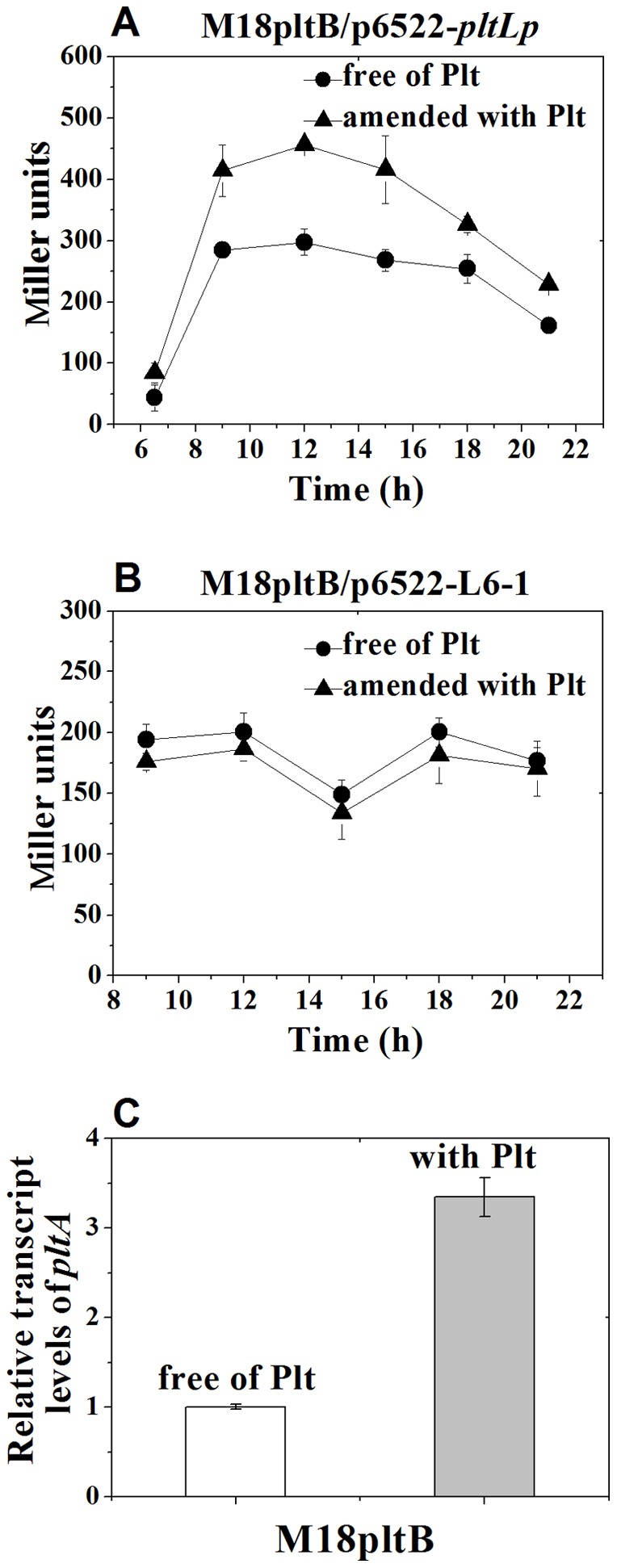
Exogenous Plt specifically induced the *pltLp* promoter expression and thus enhanced the *plt* operon transcription. (**A** and **B**) β-galactosidase expression (Miller Units) from p6522–pltLp and p6522–L6-1, which respectively carry *pltLp* (−84 bp to +1) and its upstream P_x_ promoter (+66 bp to +124 bp), was assayed in the KMB without or with 10 μg mL^−1^ exogenous Plt. The addition of 10 μg ml^−1^ exogenous Plt significantly enhanced the *pltLp–lacZ* expression (A), but did not influence *P_X_–lacZ* fusion expression (B) in the Plt-defective mutant M18pltB. (**C**) qRT-PCR analysis of the *pltA* transcript level in the strain M18pltB grown in the KMB without or with 10 μg ml^−1^ exogenous Plt. The *pltA* transcript level was significantly enhanced by the addition of exogenous Plt.

The autoinduction phenomenon, wherein Plt enhance the expression of its own biosynthetic operon *pltLABCDEFG*, has been reported in *P. fluorescens* Pf-5 [18] and *P. aeruginosa* M18 [4]. To assess whether the *pltLp* is specifically induced by Plt, two *lacZ* fusion plasmids, p6522–pltLp and p6522–L6-1, which respectively carry the *pltLp* and its upstream P_x_ promoter, were transformed into M18pltB mutant not producing Plt. β-Galactosidase activity was assayed in KMB media amended without or with 10 µg ml^−1^ exogenous Plt. As depicted in Figs. 6A and 6B, the *pltLp–lacZ* fusion expression was significantly enhanced by the addition of exogenous Plt, whereas the *P_x_–lacZ* fusion expression did not reveal any noticeable difference between the KMB with and without 10 µg ml^−1^ exogenous Plt. The data clearly suggests that the PltR-activated *pltLp* was specifically induced by the secondary metabolite Plt. Moreover, the qRT-PCR result also shows that the transcript accumulation of *pltA*, which is the second gene in the *pltLABCDEFG* biosynthetic structural operon, in the M18pltB strain was strongly increased in KMB with 10 µg ml^−1^ exogenous Plt compared with that in KMB without the addition of Plt (Fig. 6C).

## Discussion

**Figure 7:**
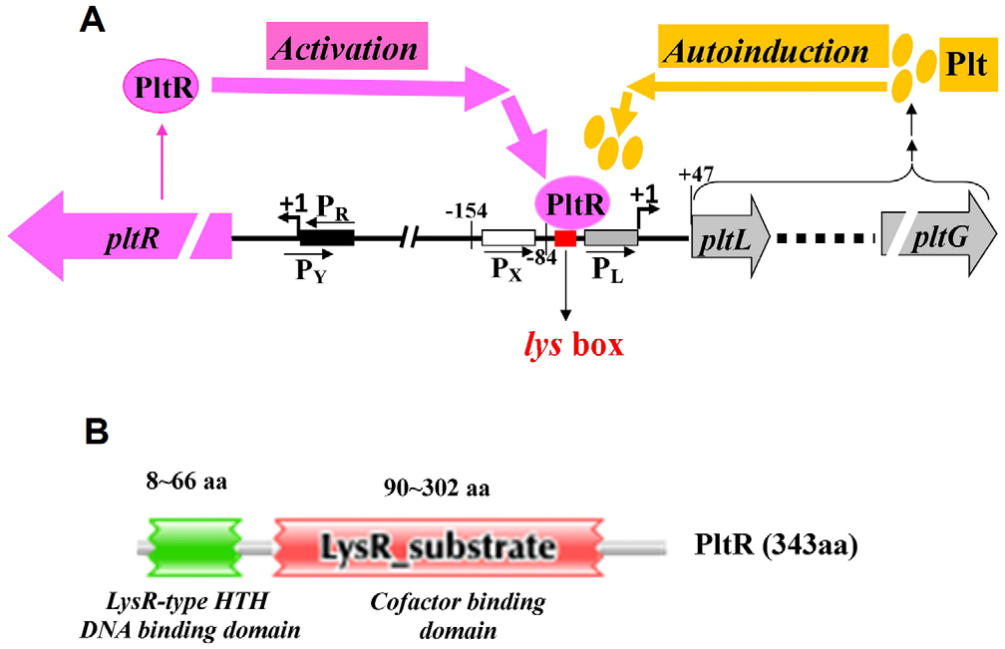
Proposed model for the transcriptional activation of PltR and autoinduction of pyoluteorin on Plt biosynthetic operon in *P. aeruginosa* M18. (**A**)**** Thick arrows indicate activation, autoinduction, and positive regulation; thin arrows indicate biogenesis. P_L_ and P_R_ denote the promoters of *pltL* and *pltR*. P_X_ and P_Y_ are two uncharacterized promoters. The TSS is shown with +1. The LysR-type regulator PltR transcriptionally activates the *plt* operon expression by directly binding to the *pltLp* region at the indispensable palindromic *lys* box. PltR also functions as a candidate receptor of Plt. Plt, as a potential and nonessential cofactor of PltR, specifically induces the expression of the *pltL* promoter and thus the *plt* operon. (**B**) **The PltR protein comprise two domains:** the N-terminal LysR-family HTH (helix-turn-helix) DNA-binding domain and the C-terminal co-inducer binding domain (predicted by Pfam 25.0). aa, amino acids.

In this study, we identified three promoters in the *pltL* direction, including the *pltL* promoter (*pltLp*) that is specifically activated by PltR and its upstream closely neighboring promoter (P_x_) that is not controlled by PltR, and one promoter (*pltR* promoter) in the *pltR* direction from the complex intergenic region between the divergently transcribed *pltR* and *pltLABCDEFG* operons (Fig. 2). PltR, a pathway-specific transcriptional activator of the *plt* operon, was shown to directly bind to the *pltLp* region at the indispensable *lys* box, a 22 bp palindromic sequence closely linking two neighboring promoters *pltLp* and P_x_. Plt, as a potential and non-essential cofactor of PltR, specifically induces the expression of the *pltLp* promoter and thus enhance Plt's own biosynthetic operon expression and biosynthesis. These are summarized in a proposed model describing transcriptional activation and autoinduction of Plt biosynthesis in *P. aeruginosa* M18 (Fig. 7A). In addition to the pathway-specific regulation, the *plt* biosynthetic operon is also subject to extensive global regulation from the Las, Rhl, and PQS QS systems [14]–[16] and the Gac/Rsm signal transduction system [12], [13].

LTTRs are known to bind mostly at the palindromic *lys* box neighboring or overlapping the upstream region of the target promoter. The *lys* box typically consists of the T-N_11_-A sequence [19]. Similarly, as a typical LTTR protein, PltR contains a DNA-binding HTH motif for the potential binding of *pltLp* and a co-inducer binding motif as the candidate binding site of Plt (Fig. 7B). The target *lys* box of PltR, a 22 bp interrupted (by 4 bp) palindromic sequence, was localized from −53 bp to −84 bp upstream of the *pltL* gene TSS. Moreover, it also carries the classic T-N_11_-A sequence. Interestingly, this 22 bp palindromic sequence was shown (by BLAST analysis) to be the only homologous sequence commonly shared by two *pltR*–*pltL* intergenic regions from *P. aeruginosa* M18 and *P. fluorescens* Pf-5 (data not shown). This phenomenon suggests that the common mechanisms involved in PltR activation and Plt autoinduction may exist between both these strains.

Studies on transcriptional activation by several members of the LTTR family have revealed many mechanistic details, and the “sliding dimer” model, a general model for inducer-dependent activation, has been proposed [19], [26]. According to this model, two binding sites, designated RBS (regulatory binding site) and ABS (activation binding site), was respectively occupied by two dimers and thus induced to form a sharp bending. However, the *pltLp* activation by PltR may not comply with the “sliding dimer” model because of the presence of the P_x_ promoter closely neighboring upstream of the *pltLp*. Intriguingly, the gradual opening of the *lys* box formed stem-loop through a 2 bp to 6 bp substitution gave rise to a significant decrease and even complete inhibition in both the *pltLp* activity and the PltR–*pltLp* binding. This decrease was not restored by the reformation of similar stem-loops via a complementary mutation of a 4 bp replacement (Figs. 4A and 5). Thus, not only secondary structure but also primary sequence of the target DNA may be critical for the specific binding of PltR–*pltLp*.

Autoinduction is widespread in gene regulation involved in bacterial secondary metabolism, such as QS signal and antibiotic biosynthesis [9], [18], [23], [25]. In contrast to that the transcriptional activator PltR mediated the induction of *plt* operon expression by Plt, the TetR-type transcriptional repressor PhlF was confirmed to mediate the induction of *phl* operon expression by 2,4-DAPG (diacetylphloroglucinol) in *P. fluorescens* CHA0 [23]. PhlF represses *phlA* transcription by binding to the *phlA* operator in *P. fluorescens* F113 [27], which in turn can be derepressed potentially through the dissociation of PhlF from the *phlA* operator by 2,4-DAPG [23]. Phloroglucinol, a precursor of 2,4-DAPG, was reported to mediate cross-talk between Plt and 2,4-DAPG biosynthetic pathways in *P. fluorescens* Pf-5 [9]. In the present study, Plt may directly bind to its candidate receptor PltR, thereby promoting the binding of PltR with the *pltLp* at the *lys* box and transcriptional activation efficiency of PltR. However, further research is needed to confirm the direct binding of Plt to PltR and to assess whether the Plt binding causes the binding enhancement and allosteric transition of the PltR–DNA complex.

Two promoters, *pltLp* and P_x_ (Fig. 2), which were not identified by the NNPP prediction program, might not carry the conserved promoter sequence. The concurrent presence of three promoters in the *pltL* direction implies that these promoters may be recognized by different sigma factors in response to different signals. An *rpoS* mutant or multiple copies of *rpoD* reportedly stimulates the overproduction of Plt and DAPG in *P. fluorescens* Pf-5 and CHA0 [28], [29] and Plt overproduction in *P. aeruginosa* M18 [30], [31]. Further research is needed to identify the TSS and function of P_x_, define the relationship between the *pltLp* and P_x_, and assess the possibility of the occurrence of sRNA within the *pltR–pltL* intergenic region. In addition, the result that the LacZ expression deriving from p6522-L1 was started in *E. coli* DH5α, whereas was entirely inhibited in *P. aeruginosa* M18 (Figs. 1C and 1D) suggests that a potential promoter (P_Y_) in the *pltL* direction is far from the neighboring *pltLp* and P_x_ promoters. The P_Y_ promoter was shown to partly overlap with the *pltR* promoter (P_R_) (Figs. 1A and 1C); they displayed similar expression in *E. coli* DH5α(Figs. 1B and 1D). However, whether the P_Y_ and P_R_ promoters constitute a bidirectional promoter remains unknown.

In summary, *pltLp* and its upstream neighboring *lys* box have been identified and characterized for PltR binding from the complicated *pltR–pltL* intergenic region. Plt, a potential and non-essential cofactor of PltR, specifically induces the *pltLp* expression and thus strengthens its own *plt* biosynthetic operon. Further work is needed to assess the possibility for the direct binding of Plt by PltR and ascertain whether the Plt binding causes the enhancement and conformation transition of PltR-*pltLp* binding and thereby promotes the transcriptional activation efficiency of PltR. In addition, we will focus on elaborating the complicated regulatory mechanisms hidden within the 652 bp *pltR–pltL* intergenic sequence, including the promoters, *cis*-elements, and potential sRNAs involved in the regulation of Plt biosynthesis in *P. aeruginosa* M18.

## Materials and Methods

### Bacterial strains, plasmids, oligonucleotides, and culture conditions

The bacterial strains, plasmids, and primers used in the present study are listed in Table 1 and Table S1. *Escherichia coli* was cultured in Luria–Bertani (LB) broth at 37°C [32]. *P. aeruginosa* M18 and its derivatives were grown in King's medium B (KMB) at 28°C [33]. When needed, 0.5 mM IPTG and 20 μg ml^−1^ X-Gal were used for blue/white screening, and 4 mg ml^−1^ ortho-nitrophenyl-β-d-galactopyranoside (ONPG) in 100 mM phosphate buffer (pH 7.0) were used for β-galactosidase assays. Antibiotics were added in the following final concentrations (μg ml^−1^): for *Pseudomonas*, spectinomycin (Sp) 100, tetracycline (Tc) 120, gentamicin (Gm) 45, kanamycin (Km) 50 and chloramphenicol (Cm) 200 for *Pseudomonas*; Tc 15, Gm 10, Km 50 and Cm 40 for *E. coli*.

**Table 1:** Bacterial strains and plasmids used in this study.

Strains/plasmids	Genotype and relevant characteristics	Reference
***E. coli***		
DH5α	*supE*44Δ*lac*U169(Φ80*lacZ*ΔM15)*hsdR*17 *recA*1 *endA*1 *gyrA*96 *thi-*1 *relA*1	
BL21 (DE3)	*E.coli B,* F- , *ompT, hsdSB(rB-mB-), gal, dcm* (DE3)	
***P. aeruginosa***		
M18WT	Wild type	Huang *et al.* [5]
M18pltR	*pltR*::Gm^r^, Sp^r^ Gm^r^	Yan *et al.* [15]
M18pltB	*pltB*::Gm^r^, Sp^r^ Gm^r^	Huang *et al.* [4]
**Plasmids**		
pME6522	pVS1-p15A *E.coli* – *Pseudomonas* shuttle vector for transcriptional *lacZ* fusions and promoter probing, Tc^r^	Blumer *et al.* [38]
p6522-R1 to R4	Four fragments, R1 to R4, which respectively harbor five putative promoters in the *pltR* direction (R3 includes two putative promoters), were fused with the upstream of promoter-less *lacZ* gene on pME6522, Tc^r^	This study
p6522-L1 to L5	Five fragments, L1 to L5, which contain five putative promoters in the *pltL* direction, were respectively fused into the upstream of *lacZ* gene in pME6522, Tc^r^	This study
p6522-L3-1 to L3-4	Four prolonging fragments based on L3, L3-1 to L3-4, was respectively fused with the *lacZ* gene in pME6522, Tc^r^	This study
P6522-L6	The L6 fragment (covering 235 bp upstream the *pltL* translational start codon) in the *pltL* direction, which was not predicted to contain promoter, was fused into the upstream of *lacZ* gene in pME6522, Tc^r^	This study
P6522-L6-1 to L6-3	Three shortened derivative fragments based on L6, L6–1 to L6–3, and the L6–4 fragment, were respectively cloned into pME6522, Tc^r^	This study
p6522-L6-3-1 to L6-3-4	Five continuously prolonged fragments based on L6–3, L6-3-1 to L6-3-4, were respectively cloned into pME6522, Tc^r^	This study
p6522-pltLp	The 85 bp *pltL* promoter fragment was cloned into pME6522, Tc^r^	This study
p6522-pltLp-2 to pltLp-3	Two mutagenized *pltL* promoter fragments (64 bp) with a half or entire deletion of palindromic *lys* box were respectively cloned into pME6522, Tc^r^	This study
p6522-pltLp-M2 to pltLp-M6	Three mutant fragments of *pltLp* promoter, *pltLp-M2*, *-M4*, and *-M6*, which respectively carry 2-bp, 4-bp, and 6-bp replacement in the *lys* box, were cloned into pME6522, Tc^r^	This study
P6522-pltLp-M4-M	The *pltLp-M4-M* fragment, which was generated by introducing a 4-bp complementary replacement in the *lys* box into the *pltLp-M4* mutant, was cloned into pME6522; Tc^r^	This study
pBBR1MCS	Cloning vector, broad host range, IncP IncQ, Cm^r^	Kovach *et al.* [39]
pBBR-pltR	A 1198 bp *BamH*I-*Hind*III fragment containing the 1032 bp entire ORF of *pltR* and its upstream promoter/operator region was cloned into pBBR1MCS, Cm^r^	This study
pET28a	T7 expression vector, Km^r^	Novagen
pET-pltR	A 1052 bp *Nde*I-*Hind*III fragment containing the 1032 bp entire ORF of *pltR* was cloned into pET28a, Km^r^	This study

### DNA manipulation and analyses

All molecular biology methods not depicted in detail were carried out using standard procedures [32]. General molecular biological reagents or kits were used according to the manufacturers' specifications, including Taq, LA Taq (TaKaRa), and KOD *plus* DNA polymerase (Toyobo), restriction endonucleases, DNA ligase, DNA and protein markers, plasmid DNA (TaKaRa or BioDev) and genomic DNA extraction kits (BBI), and DNA gel recovery kits (Qiagen or Axygen). DNA was synthesized by Shanghai Sangon Biological Engineering Technology and Services Co., Ltd., and sequenced by the Invitrogen Biotechnology Corporation and the Beijing HuaDa Genomics Institute.

Neural Network Promoter Prediction (NNPP) software (v. 2.2) was used to identify putative promoter elements of the *pltR*–*pltL* intergenic region, with a minimum promoter score of 0.8 [34]. The secondary structure of the *pltLp* sequence was predicted by Mfold 3.2 at 28°C. The most stable structure was chosen when multiple structures were generated [35]. The probable protein domain analysis of PltR was performed using Pfam [36].

### 5′Rapid amplification of cDNA end (5′RACE) for identifying the transcriptional start sites (TSS) of *pltR* and *pltLABCDEFG*

5′RACE was performed using an Invitrogen 5′RACE system. Total RNAs were isolated using the NucleoSpin® RNA II kit (Macherey–Nagel). The first strand cDNAs of *pltR* and *pltL* were respectively synthesized from total RNA templates using DNA specific primers Ptsr1 and Ptsl1, and purified with a S.N.A.P column. A homopolymeric C tail was then added to the 3′-end of cDNAs using deoxynucleotidyl transferase (TdT) and dCTP. PCR amplification of the dC-tailed cDNA was carried out using the novel deoxyinosine-containing abridged anchor primer (AAP) and the nested DNA specific primers Ptsr2 or Ptsl2. The PCR products were purified and cloned into the pMD18-T vectors (TaKaRa). The TSSs were identified through the sequencing of multiple independent clones.

### Construction of *lacZ* fusion plasmids and β-galactosidase activity assay

The reporter plasmid pME6522, which carries a promoterless *E. coli lacZ* gene downstream from a multiple cloning site and encodes Tc resistance, was used to probe unknown promoter activity or construct transcriptional fusion plasmid. As shown in Figs. 1A and 1C, two sets of DNA fragments containing all putative promoters (predicted by NNPP, *P*>0.8) at the 5′-terminals of *pltR* and *pltL* operons were respectively amplified and cloned into the *Eco*RI/*Pst*I-digested pME6522 to identify actual promoter activity. Four fragments (L3–1 to L3–4), which extend continuously from the right of the L3 fragment, were cloned into pME6522 to probe for potential promoter activity and location within the L6 fragment not predicted to carry any promoters of the *pltLABCDEFG* operon (Fig. 1E). The L6 fragment and its eight systematically deleted fragments were inserted into the plasmid pME6522 to analyze the *cis-*regulatory elements in the 5′-leader of the *plt* operon, including promoters and the PltR's acting site (Fig. 1G).

The *pltLp–lacZ* transcriptional fusion plasmid (p6522–pltLp) was constructed by introducing a 85 bp DNA fragment, which ranges from −84 bp to the actual TSS (+1) and harbors the *pltLp* and its upstream neighboring palindromic *lys* box (Fig. 2A), into the plasmid pME6522, thereby generating p6522–pltLp (Fig. 3A and 4B). The *pltLp* fragments with deletion of half or the entire *lys* box were cloned into pME6522, generating two fusion plasmids, p6522–pltLp-2 and p6522–pltLp-3 (Fig. 4B). All recombined *lacZ* reporter plasmids in this study have been verified by sequencing with a *lacZ* primer.

*P. aeruginosa* M18 and its derivative strains carrying the *lacZ* reporter plasmids above were inoculated from the overnight culture into 500-ml Erlenmeyer flasks containing 100 ml KMB media to a final concentration of OD_600_  = 0.05, and grown at 28°C with shaking at 200 rpm. β-Galactosidase activities were measured according to the method by Miller [37] and as described by Huang *et al.* [5].

### qRT-PCR

*P. aeruginosa* M18 and its derivative mutant M18pltB were grown in KMB with or without 10 µg ml^−1^ Plt. Cells were harvested from 0.5 ml of cultures with an OD_600_ of 3.5 to 5 and submitted to quantify the accumulation level of *pltA* mRNA by quantitative reverse transcription PCR (qRT-PCR). Total RNA was isolated with NucleoSpin RNA II kit (Macherey–Nagel) and treated with RQ1 RNase-free DNase (Promega). First strand cDNA was synthesized using RevertAid™ First Strand cDNA Synthesis Kit (MBI Fermentas) and quantified using a RealMasterMix (SYBR Green) RT-PCR Kit (Tiangen). The qRT-PCR amplicons of *pltA* (157 bp) and the reference gene *rpoD* (173 bp) were synthesized from their first strand cDNA templates with the corresponding gene-specific primers. The results were normalized using the constitutively expressed gene *rpoD* as the internal control. Experiments were carried out in triplicate.

### Overexpression and purification of PltR

An 1178 bp fragment, including the entire *pltR* gene (1032 bp) and its operator/promoter region (146 bp), was amplified and cloned into the *E. coli–Pseudomonas* shuttle vector pBBR1MCS at the *Bam*HI and *Hind*III sites, thus constructing the *pltR* overexpression plasmid pBBR–pltR.

The entire *pltR* ORF (1032 bp) was amplified and inserted into the *Nde*I/*Bam*HI-digested pET28a plasmid. The resulting plasmid, pET–pltR, was transformed into *E. coli* BL21 for overexpression and purification of the C-terminus His_6_–tagged PltR protein. *E. coli* BL21 carrying the pET28a–*pltR* plasmid was grown at 37°C in 1000 ml LB media, with shaking to an OD_600_ of 0.8, and induced with 0.5 mM IPTG. Subsequently, cell cultures were incubated at 16°C for 16 h. The cells were harvested by centrifugation and resuspended in 10 ml nickel A buffer (20 mM imidazole, 300 mM NaCl, 25 mM Tris–HCl at pH 8.0). The resuspending solution was added 1 μg ml^−1^ leupeptin, 1 μg ml^−1^ aprotinin, and 50 μM phenylmethylsulfonyl fluoride (PMSF) and shaken at 4°C for 30 min. The cells were lysed in ultrasonic cell disruptor. The protein extract was loaded onto a Ni–NTA agarose column (GE healthcare) and washed twice with 5 ml nickel buffer. The PltR protein was eluted with the elution buffer containing 500 mM imidazole. The purified PltR protein was stored in a buffer containing 20 mM Tris–HCl (pH 8.0), 200 mM NaCl, 1 mM DTT, and 1 mM EDTA at −80°C after removing the imidazole by HPLC (AKTA).

### Electrophoretic mobility shift assay (EMSA) of DNA–protein interaction

DNA probes were generated by PCR with the corresponding biotin-labeled primers (Table S1). EMSA was performed using a Thermo Scientific LightShift Chemiluminescent EMSA Kit (No. 20148). A total of 0.5 nM biotin-labeled DNA probes were incubated with increasing concentrations of PltR protein in 10 ul binding buffer. The reaction mixtures were incubated for 20 min to 40 min at room temperature. Subsequently, 6 ul of the samples were separated by electrophoresis on 6% native polyacrylamide gel and electrotransferred to a positively charged nylon membrane (Ambion). The transferred PltR–DNA complexes and free DNA probes were cross-linked to the membrane for 2 min with a 320 nm UV light cross-linking instrument. Finally, the biotin-labeled bands were detected using a Thermo Scientific Chemiluminescent Nucleic Acid Detection Module.

### Site-directed mutagenesis of the 22 bp palindromic *lys* box

Three mutants of the *pltLp* region (−84 bp to +1), which respectively carried 2 bp (M2: G74T, C73A), 4 bp (M4: G74T, C73A, T71G, T70C), and 6 bp replacements (M6: G74T, C73A, T71G, T70C, G68A, C67T) in the *lys* box (Fig. 5A), were generated by PCR amplification with the respective mutant forward primer and the same non- or biotin-labeled reverse primer (Table S1). Similarly, a 4 bp complementary replacement was introduced to the M4 mutant to construct the M4-M (G74T, C73A, T71G, T70C, and A57G, A56C, G54T, C53A) *pltLp* mutant (Fig. 5A). The mutant PCR products were cloned into the *Eco*RI/*Pst*I-digested pME6522, producing the corresponding *lacZ* fusion reporter plasmids. In addition, the biotin-labeled mutant PCR products were used for the EMSA analysis.

### Effect of exogenous Plt on the *pltLp* and *plt* operon expression

The *pltB* mutant M18pltB carrying the *lacZ* reporter plasmid p6522–pltLp or p6522–L6-1 was grown in KMB with or without Plt at a final concentration of 10 μg ml^−1^ (dissolved in methanol). β-Galactosidase activity was measured to confirm the specific induction of *pltLp* by Plt. Similarly, the *pltA* mRNA level of the M18pltB strain was assayed by qRT-PCR in KMB with or without 10 μg ml^–1^ Plt.

## Supporting Information

Table S1 **Primers used in this study.**
(DOC)

## References

[1] Bender C, Rangaswamy V, Loper J (1999). Polyketide production by plant-associated *Pseudomonads*.. Annu Rev Phytopathol.

[2] Brodhagen M, Paulsen I, Loper JE (2005). Reciprocal regulation of pyoluteorin production with membrane transporter gene expression in *Pseudomonas fluorescens* Pf-5.. Appl Environ Microbiol.

[3] Nowak-Thompson B, Chaney N, Wing JS, Gould SJ, Loper JE (1999). Characterization of the pyoluteorin biosynthetic gene cluster of *Pseudomonas fluorescens* Pf-5.. J Bacteriol.

[4] Huang X, Yan A, Zhang X, Xu Y (2006). Identification and characterization of a putative ABC transporter PltHIJKN required for pyoluteorin production in *Pseudomonas* sp. M18.. Gene.

[5] Huang X, Zhu D, Ge Y, Hu H, Zhang X (2004). Identification and characterization of *pltZ*, a gene involved in the repression of pyoluteorin biosynthesis in *Pseudomonas* sp. M18.. FEMS Microbiol Lett.

[6] Winstanley C, Langille MG, Fothergill JL, Kukavica-Ibrulj I, Paradis-Bleau C (2009). Newly introduced genomic prophage islands are critical determinants of in vivo competitiveness in the Liverpool Epidemic Strain of *Pseudomonas aeruginosa*.. Genome Res.

[7] Wu DQ, Ye J, Ou HY, Wei X, Huang X (2011). Genomic analysis and temperature-dependent transcriptome profiles of the rhizosphere originating strain *Pseudomonas aeruginosa* M18.. BMC Genomics.

[8] Paulsen IT, Press CM, Ravel J, Kobayashi DY, Myers GS (2005). Complete genome sequence of the plant commensal *Pseudomonas fluorescens* Pf-5.. Nat Biotechnol.

[9] Kidarsa TA, Goebel NC, Zabriskie TM, Loper JE (2011). Phloroglucinol mediates cross-talk between the pyoluteorin and 2,4-diacetylphloroglucinol biosynthetic pathways in *Pseudomonas fluorescens* Pf-5.. Mol Microbiol.

[10] Haas D, Keel C (2003). Regulation of antibiotic production in root-colonizing *Pseudomonas spp.* and relevance for biological control of plant disease.. Ann Rev Phytopathol.

[11] Lapouge K, Schubert M, Allain FHT, Haas D (2008). Gac/Rsm signal transduction pathway of gamma-proteobacteria: from RNA recognition to regulation of social behaviour.. Mol Microbiol.

[12] Zhang X, Wang S, Geng H, Ge Y, Huang X (2005). Differential regulation of *rsmA* gene on biosynthesis of pyoluteorin and phenazine-1-carboxylic acid in *Pseudomonas* sp. M18.. W J Microbiol Biotechnol.

[13] Ge Y, Huang X, Wang S, Zhang X, Xu Y (2004). Phenazine-1-carboxylic acid is negatively regulated and pyoluteorin positively regulated by *gacA* in *Pseudomonas* sp. M18.. FEMS Microbiol Lett.

[14] Lu J, Huang X, Li K, Li S, Zhang M (2009). LysR family transcriptional regulator PqsR as repressor of pyoluteorin biosynthesis and activator of phenazine-1-carboxylic acid biosynthesis in *Pseudomonas* sp. M18.. J Biotechnol.

[15] Yan A, Huang X, Liu H, Dong D, Zhang D (2007). An *rhl*-like quorum-sensing system negatively regulates pyoluteorin production in *Pseudomonas* sp. M18.. Microbiology.

[16] Chen Y, Wang X, Huang X, Zhang X, Xu Y (2008). Las-like quorum-sensing system negatively regulates both pyoluteorin and phenazine-1-carboxylic acid production in *Pseudomonas* sp. M18.. Sci China C Life Sci.

[17] Yan A, Wang X, Zhang X, Xu Y (2007). LysR family factor PltR positively regulates pyoluteorin production in a pathway-specific manner in *Pseudomonas* sp. M18.. Sci China Ser C-Life Sci.

[18] Brodhagen M, Henkels MD, Loper JE (2004). Positive autoregulation and signaling properties of pyoluteorin, an antibiotic produced by the biological control organism *Pseudomonas fluorescens* Pf-5.. Appl Environ Microbiol.

[19] Maddocks SE, Oyston PC (2008). Structure and function of the LysR-type transcriptional regulator (LTTR) family proteins.. Microbiology.

[20] Schell MA (1993). Molecular biology of the LysR family of transcriptional regulators.. Annu Rev Microbiol.

[21] Xiao GP, Deziel E, He JX, Lepine F, Lesic B (2006). MvfR, a key *Pseudomonas aeruginosa* pathogenicity LTTR-class regulatory protein, has dual ligands.. Mol Microbiol.

[22] Venturi V (2006). Regulation of quorum sensing in *Pseudomonas*.. FEMS Microbiol Rev.

[23] Schnider-Keel U, Seematter A, Maurhofer M, Blumer C, Duffy B (2000). Autoinduction of 2,4-diacetylphloroglucinol biosynthesis in the biocontrol agent *Pseudomonas fluorescens* CHA0 and repression by the bacterial metabolites salicylate and pyoluteorin.. J Bacteriol.

[24] Li Y, Du X, Lu ZJ, Wu D, Zhao Y (2011). Regulatory feedbackloop of two *phz* gene clusters through 5'-untranslated regions in *Pseudomonas* sp. M18.. PLoS One.

[25] Lamont IL, Beare PA, Ochsner U, Vasil AI, Vasil ML (2002). Siderophore-mediated signaling regulates virulence factor production in *Pseudomonas aeruginosa*.. Proc Natl Acad Sci U S A.

[26] Porrua O, Platero AI, Santero E, del Solar G, Govantes F (2010). Complex interplay between the LysR-type regulator AtzR and its binding site mediates *atzDEF* activation in response to two distinct signals.. Molecular Microbiology.

[27] Abbas A, Morrissey JP, Marquez PC, Sheehan MM, Delany IR (2002). Characterization of interactions between the transcriptional repressor PhlF and its binding site at the phlA promoter in *Pseudomonas fluorescens* F113.. J Bacteriol.

[28] Sarniguet A, Kraus J, Henkels MD, Muehlchen AM, Loper JE (1995). The sigma factor sigma s affects antibiotic production and biological control activity of *Pseudomonas fluorescens* Pf-5.. Proc Natl Acad Sci U S A.

[29] Schnider U, Keel C, Blumer C, Troxler J, Defago G (1995). Amplification of the housekeeping sigma factor in Pseudomonas fluorescens CHA0 enhances antibiotic production and improves biocontrol abilities.. J Bacteriol.

[30] Ge Y, Pei D, Li W, Zhao Y, Xu Y (2006). Insertional mutation of the *rpoS* gene contributes to alteration in biosynthesis of antifungal agents in *Pseudomonas* sp. M18.. Biol Control.

[31] Zhu D, Xu W, Geng H, Zhang X, Xu Y (2003). Gene cloning of *rpoD* and its impact on biosynthesis of antibiotics in fluorescent *Pseudomonas* M18.. Wei Sheng Wu Xue Bao.

[32] Sambrook J, Russell DW (2001). Cold Spring Harbor: CSH Press..

[33] King EO, Ward MK, Raney DE (1954). Two simple media for the demonstration of pyocyanin and fluorescin.. J Lab Clin Med.

[34] Reese MG (2001). Application of a time-delay neural network to promoter annotation in the *Drosophila melanogaster* genome.. Comput Chem.

[35] Zuker M (2003). Mfold web server for nucleic acid folding and hybridization prediction.. Nucleic Acids Res.

[36] Bateman A, Birney E, Cerruti L, Durbin R, Etwiller L (2002). The Pfam protein families database.. Nucleic Acids Res.

[37] Miller JH (1972). Experiments in Molecular Genetics. Cold Spring Harbor, N.Y., USA.. : Cold Spring Harbor Laboratory Press.

[38] Blumer C, Heeb S, Pessi G, Haas D (1999). Global GacA-steered control of cyanide and exoprotease production in *Pseudomonas fluorescens* involves specific ribosome binding sites.. Proc Natl Acad Sci U S A.

[39] Kovach ME, Phillips RW, Elzer PH, Roop RM, 2nd, Peterson KM (1994). pBBR1MCS: a broad-host-range cloning vector.. Biotechniques.

