# Supplementary material for: Transcriptional Activation of Pyoluteorin Operon Mediated by the LysR-Type Regulator PltR Bound at a 22 bp lys Box in Pseudomonas aeruginosa M18
Source: PLoS One. 2012 Jun 25;7(6):e39538. doi: 10.1371/journal.pone.0039538 (PMC3382589; doi:10.1371/journal.pone.0039538)
Supplement: Table S1 — Primers used in this study. (DOC) [file pone.0039538.s001.doc]

**Table S1. Primers used in this study**

| ***Gene*** | ***Length(nt)*** | | | ***Primer sequence*** | |
| --- | --- | --- | --- | --- | --- |
| **5´RACE** | | | | | |
| *pltR* | | 448 | Ptsr1: 5'-AGAAATCCAGTTTGAGGC-3' | | |
| *pltR* | | 303 | Ptsr2: 5'-GTTGATGTTTCTCGGAATT-3' | | |
| *pltL* | | 434 | Ptsl1: 5'- CACTCCCATTTCATCCCT -3' | | |
| *pltL* | | 328 | Ptsl2: 5'- ACACCATCACCGACAAGG-3' | | |
| **Construction of *lacZ* fusion plasmid** | | | | | |
| L1 | | 164 | P101: 5'-CGCG***GAATTC***TCCGAAAAACTACCG-3' (*EcoR*I) | | |
|  | |  | P102: 5'-TGGC***CTGCAG***CATCCTTCCATGATG-3' (*Pst*I) | | |
| L2 | | 121 | P103: 5'- CGCG***GAATTC***TCAAGAGCGAGGTGT-3' (*EcoR*I) | | |
|  | |  | P104: 5'-AGTC***CTGCAG***GTTACGTCCGGCTAT-3' (*Pst*I) | | |
| L3 | | 194 | P105: 5'- CGCG***GAATTC***CAGGAGAGTCGATTC-3' (*EcoR*I) | | |
|  | |  | P106: 5'- ATAT***CTGCAG***CCGGACGACCTGCTA-3' (*Pst*I) | | |
| L4 | | 160 | P107: 5'- CGCG***CTGCAG***ATGGATATGGAAGAG-3' (*Pst*I) | | |
|  | |  | P108: 5'- ATAT***CTGCAG***CCCTGACGAAAACTA-3' (*Pst*I) | | |
| L5 | | 119 | P109: 5'- CGCG***GAATTC***TAGTTTTCGTCAGGG-3' (*EcoR*I) | | |
|  | |  | P110: 5'- ATAT***CTGCAG***AGCCGGTTCGGCATT-3' (*Pst*I) | | |
| L6 | | 235 | P111: 5'-CGCG***GAATTC***CAGCCTCTTCAGGGAA-3' (*EcoR*I) | | |
|  | |  | P112: 5'- TGTC***CTGCAG***CACGTCCCCTAGAA-3' (*Pst*I) | | |
| R1 | | 198 | P201: 5'- AGCG***GAATTC***ATACGGCGCACGAGA-3' (*EcoR*I) | | |
|  | |  | P202: 5'- GCAT***CTGCAG***GACGCACATAACTGG-3' (*Pst*I) | | |
| R2 | | 182 | P203: 5’- AGCG***GAATTC***CGGAGTATCGTCCTG-3’ (*EcoR*I) | | |
|  | |  | P204: 5'- GCGC***CTGCAG***CTATTCAAATTCCAA-3' (*Pst*I) | | |
| R3 | | 204 | P205: 5'- GCGC***GAATTC***CTTGACAATGACTAC-3' (*EcoR*I) | | |
|  | |  | P206: 5'- CTAT***CTGCAG***TGAAACCGAAACCGG-3' (*Pst*I) | | |
| R4 | | 126 | P207: 5'- GCGC***GAATTC***TGACTTTCCAAGGTC-3' (*EcoR*I) | | |
|  | |  | P208: 5'- CTAT***CTGCAG***GCGATTCGCTATCCC-3' (*Pst*I) | | |
| L3-1 | | 250 | P105 | | |
|  | |  | PL3-1 down: 5'-TATG***CTGCAG***CGCTGCAAGCAATGG-3' (*Pst*I) | | |
| L3-2 | | 308 | P105 | | |
|  | |  | PL3-2 down: 5'-ACGTT***CTGCAG***GGTACGGGGACTAT-3' (*Pst*I) | | |
| L3-3 | | 389 | P105 | | |
|  | |  | PL3-3 down: 5'-CATCC***CTGCAG***CAACCTTCCACGAGA-3' (*Pst*I) | | |
| L3-4 | | 437 | P105 | | |
|  | |  | P112 | | |
| L6-1 | | 106 | P111 | | |
|  | |  | PL3-2 down | | |
| L6-2 | | 85 | PL6-2 up: 5'-ATATA***GAATTC***AGCTCCCGCATGCCA-3' (*EcoR*I) | | |
|  | |  | PL3-2 down | | |
| L6-3 | | 59 | PL6-3 up: 5'-ATAC***GAATTC***GGCTTGGACGGTTGC-3' (*EcoR*I) | | |
|  | |  | PL3-2 down | | |
| L6-3-1 | | 78 | PL6-3 up | | |
|  | |  | PL6-3 down: 5'-ATGC***CTGCAG***CGCAAAGGCGCTCTA-3' (*Pst*I) | | |
| L6-3-2 | | 92 | PL6-3 up | | |
|  | |  | PL6-3-2 down: 5'-ATAT***CTGCAG***GGCCTTTGCGGCTGC-3' (*Pst*I) | | |
| L6-3-3 | | 119 | PL6-3 up | | |
|  | |  | PL6-3-3 down: 5'-GCGC***CTGCAG***GGAATTTGAATAGCG-3' (*Pst*I) | | |
| L6-3-4 | | 140 | PL6-3 up | | |
|  | |  | PL3-3 down | | |
| L6-4 | | 82 | PL6-4 up: 5'-GTAG***GAATTC***GTCCTAGAGCGCCT-3' (*EcoR*I) | | |
|  | |  | PL3-3 down | | |
| *pltLp* | | 85 | PL6-4 up | | |
|  | |  | PpltLp down: 5'-GACG***CTGCAG***CAGCCAACCTTCCACGAGAA  CA-3' (*Pst*I) | | |
| *pltLp-2* | | 64 | PpltLp-2 up: 5'-AT***GAATTC***GCCGCAAAGGCCTTTTGAGGAT-3' (*EcoR*I) | | |
|  | |  | PpltLp down | | |
| *pltLp-3* | | 64 | PpltLp-3 up: 5'-GGCT***GAATTC***GCCTTTTGCGGATTG-3' (*EcoR*I) | | |
|  | |  | PpltLp-3 down: 5'-GCGT***CTGCAG***TTAAATTGCCAGCCA-3' (*Pst*I) | | |
| *pltLp-M2* | | 85 | PpltLp-M2 up:5'-AT***GAATT***CGTCCTAGAGCTACTTTGCGCAG  CCGCAAAG-3' (*EcoR*I) | | |
|  | |  | PpltLp-M down:5'-ACTGATGA***CTGCAG***CAGCCAACCTTCCAC  GAGAACATT-3' (*Pst*I) | | |
| *pltLp-M4* | | 85 | PpltLp-M4 up:5'-AT***GAATT***CGTCCTAGAGCTACGCTGCGCAG  CCGCAAA-3' (*EcoR*I) | | |
|  | |  | PpltLp-M down | | |
| *pltLp-M6* | | 85 | PpltLp-M6 up:5'-AT***GAATTC***GTCCTAGAGCTACGCTATGCAG  CCGCAAAG-3' (*EcoR*I) | | |
|  | |  | PpltLp-M down | | |
| *pltLp-M4-M* | | 85 | PpltLp-M4-M up:5'-AT***GAATTC***GTCCTAGAGCTACGCTGCG  CAGCCGCAGCGTACTT-3' (*EcoR*I) | | |
|  | |  | PpltLp-M down | | |
| **Overexpression of *pltR*** | | | | | |
| *pltR*(pBBR1MCS) | | 1178 | PpltR1: 5'-ATGC***GGATCC***TCGAATGACGGGGTA-3' (*BamH*I) | | |
|  | |  | PpltR2: 5'-GCGC***AAGCTT***TCATGAACTAACCCG-3' (*Hind*III) | | |
| *pltR*(pET28a) | | 1032 | PpltR3: 5'-GGCGG***CATATG***ATGAAAACACTTGGAT-3' (*Nde*I) | | |
|  | |  | PpltR4: 5'-AGTCA***GGATCC***TCATGAACTAACCCG-3' (*BamH*I) | | |
| **RT-PCR** | | | | | |
| *pltA* | | 157 | PpltA-R1: 5'-ACCGAGACCATCACCAGCATC-3' | | |
|  | |  | PpltA-R2: 5'-CCTTCTTGAACGGACGCACC-3' | | |
| *rpoD* | | 173 | PrpoD-R1: 5'-GAGCGGGAGGAGCGTTTAC-3' | | |
|  | |  | PrpoD-R2: 5'-CGGGCAAAAAATAAGCAGAGG-3' | | |
| **Biotin labeled DNA probes for EMSA** | | | | | |
| *pltLp-Bio* | | 85 | PpltLp up | | PpltLp down-Bio |
| *pltLp-2-Bio* | | 64 | PpltLp-2 up | | PpltLp-2 down-Bio |
| *pltLp-3-Bio* | | 54 | PpltLp-3up | | PpltLp-3 down-Bio |
| *pltLp-M2-Bio* | | 85 | PpltLp-M2 up | | PpltLp-M down-Bio |
| *pltLp-M4-Bio* | | 85 | PpltLp-M4 up | | PpltLp-M down-Bio |
| *pltLp-M6-Bio* | | 85 | PpltLp-M6 up | | PpltLp-M down-Bio |
| *pltLp-M4-M-Bio* | | 85 | PpltLp-M4-M up | | PpltLp-M down-Bio |
